# Perfluorochemicals and Human Semen Quality: The LIFE Study

**DOI:** 10.1289/ehp.1307621

**Published:** 2014-08-15

**Authors:** Germaine M. Buck Louis, Zhen Chen, Enrique F. Schisterman, Sungduk Kim, Anne M. Sweeney, Rajeshwari Sundaram, Courtney D. Lynch, Robert E. Gore-Langton, Dana Boyd Barr

**Affiliations:** 1 Division of Intramural Population Health Research, *Eunice Kennedy Shriver* National Institute of Child Health and Human Development, National Institutes of Health, Department of Health and Human Services, Rockville, Maryland, USA; 2 Department of Epidemiology and Biostatistics, University of Texas Rural School of Public Health, College Station, Texas, USA; 3 Department of Obstetrics and Gynecology, College of Medicine, The Ohio State University, Columbus, Ohio, USA; 4 The EMMES Corporation, Rockville, Maryland, USA; 5 Rollins School of Public Health, Emory University, Atlanta, Georgia, USA

## Abstract

Background: The relation between persistent environmental chemicals and semen quality is evolving, although limited data exist for men recruited from general populations.

Objectives: We examined the relation between perfluorinated chemicals (PFCs) and semen quality among 501 male partners of couples planning pregnancy.

Methods: Using population-based sampling strategies, we recruited 501 couples discontinuing contraception from two U.S. geographic regions from 2005 through 2009. Baseline interviews and anthropometric assessments were conducted, followed by blood collection for the quantification of seven serum PFCs (perfluorosulfonates, perfluorocarboxylates, and perfluorosulfonamides) using tandem mass spectrometry. Men collected a baseline semen sample and another approximately 1 month later. Semen samples were shipped with freezer packs, and analyses were performed on the day after collection. We used linear regression to estimate the difference in each semen parameter associated with a one unit increase in the natural log–transformed PFC concentration after adjusting for confounders and modeling repeated semen samples. Sensitivity analyses included optimal Box-Cox transformation of semen quality end points.

Results: Six PFCs [2-(*N*-methyl-perfluorooctane sulfonamido) acetate (Me-PFOSA-AcOH), perfluorodecanoate (PFDeA), perfluorononanoate (PFNA), perfluorooctane sulfonamide (PFOSA), perfluorooctane sulfonate (PFOS), and perfluorooctanoic acid (PFOA)] were associated with 17 semen quality end points before Box-Cox transformation. PFOSA was associated with smaller sperm head area and perimeter, a lower percentage of DNA stainability, and a higher percentage of bicephalic and immature sperm. PFDeA, PFNA, PFOA, and PFOS were associated with a lower percentage of sperm with coiled tails.

Conclusions: Select PFCs were associated with certain semen end points, with the most significant associations observed for PFOSA but with results in varying directions.

Citation: Buck Louis GM, Chen Z, Schisterman EF, Kim S, Sweeney AM, Sundaram R, Lynch CD, Gore-Langton RE, Barr DB. 2015. Perfluorochemicals and human semen quality: the LIFE Study. Environ Health Perspect 123:57–63; http://dx.doi.org/10.1289/ehp.1307621

## Introduction

Perfluorochemicals (PFCs) are a group of synthetic chemicals that have been used in many consumer products [Agency for Toxic Substances and Disease Registry (ATSDR) 2009]. The two highest-production PFCs in the United States are perfluorooctanesulfonic acid (PFOS) and perfluorooctanoic acid (PFOA), both of which are frequently detected in humans (ATSDR 2009; Kato et al. 2011). Other PFCs include perfluorohexane sulfonic acid (PFHxS), which is a member of the same chemical category as PFOS, and perfluorononanoic acid (PFNA), which is a member of the same chemical category as PFOA (U.S. Environmental Protection Agency 2009). Chemicals within a given PFC chemical category share similar chemical structures, making them stable and suitable for surface coating and protectant formulations for paper-packaging products, carpets, leather products, and textiles that repel water, grease, and soil among other uses (ATSDR 2009). Varying (in)direct sources of environmental exposure serve as routes for human exposure (Prevedouros et al. 2006) including ingestion of food and water, inhalation, and lactational transfer (Fromme et al. 2009). Two recent studies pointed to food consumption as the primary pathway of exposure to PFOS and PFOA (Kelly et al. 2009; Trudel et al. 2008), with an estimated daily uptake from food of 2–3 ng/kg (Fromme et al. 2009).

Because of their long half-lives, ranging from 3.5 to 7.3 years (Olsen et al. 2007), some PFCs remain in the environment and bioconcentrate in animals (Conder et al. 2008; Fromme et al. 2009; Kelly et al. 2007, 2009; Lau et al. 2007). PFCs are not lipophilic, but they do bind to serum albumin (Han et al. 2003), which facilitates their measurement in serum and is thus indicative of long-term exposure (Fromme et al. 2009).

For the most part, well-designed epidemiologic research focusing on environmentally relevant concentrations of PFCs and human fecundity—the biologic capacity of men and women for reproduction (Buck Louis 2011)—has begun only recently. This lack of human research is in contrast to an evolving body of evidence in experimental animals that suggests altered male fecundity (decreased testosterone and increased estradiol levels in serum) in exposed rats and lower serum testosterone concentrations and epididymal sperm counts in exposed mice (Biegel et al. 1995; Shi et al. 2007; Wan et al. 2011). However, not all animal studies have reported evidence of adverse effects (Luebker et al. 2005).

Equivocal results have emerged from three distinct samples of men in whom selected PFCs were quantified in serum or plasma along with varying semen analyses: men from the general Danish population (Joensen et al. 2009, 2013), male partners of pregnant women (Specht et al. 2012; Toft et al. 2012), and couples seeking infertility treatment (Raymer et al. 2012). Joensen et al. (2009) observed negative associations for the highest PFOA and PFOS concentrations relative to the lowest and for the median number of normal spermatozoa in the general Danish population. Toft et al. (2012) reported negative associations for an increasing percentage of defect in sperm cell morphology in relation to serum PFOS concentrations among male partners of pregnant women from two European countries, but not among Inuit men, all of whom participated in the INUENDO Study. No associations were reported by Specht et al. (2012) for serum PFOA, PFOS, PFHxS, or PFNA and DNA damage or apoptotic sperm cells in men from the INUENDO Study. In a recent cross-sectional study, Raymer et al. (2012) reported no significant associations of plasma and semen PFOS and PFOA concentrations with reproductive hormones or select semen quality end points among 256 men attending infertility clinics; these authors did not consider either morphology or DNA fragmentation. In a study involving 247 young men being considered for military service in Denmark, Joensen et al. (2013) reported only one statistically significant negative association between perfluoro-1-heptanesulfonate and progressively motile sperm, although they did observe several negative relations between PFOS and serum total and free testosterone, free androgen index and other hormonal ratios (i.e., testosterone/luteinizing hormone; testosterone/estradiol; free testosterone/luteinizing hormone; free androgen index/luteinizing hormone).

This existing body of evidence is largely limited to assessment of a few PFCs (i.e., PFOA and PFOS) in male partners of pregnant women or of couples seeking infertility treatment. To our knowledge, there has been no attempt to assess PFCs in relation to a wide range of semen quality parameters among men from the general population, in particular in the United States. Thus, we examined the relation between PFCs and semen quality among 501 male partners of couples planning pregnancy.

## Methods

*Study design and cohort*. We used the LIFE Study cohort for assessing seven PFCs in relation to 35 semen quality parameters in an attempt to explore possible associations. Briefly, 501 couples discontinuing contraception for the purposes of becoming pregnant were recruited from 16 counties in Michigan and Texas. Given the absence of established population-based sampling frameworks for identifying couples planning pregnancy (Buck et al. 2004), we utilized a marketing database in Michigan and the fishing/hunting license registry in Texas to ensure a sufficiently large denominator; couples planning pregnancy have been estimated to make up approximately 1% of the population (Buck et al. 2004; Slama et al. 2006). Forty-two percent of eligible couples enrolled in the study, as described elsewhere (Buck Louis et al. 2011). Inclusion criteria were minimal: Male partners needed to be at least 18 years of age, in a committed relationship, and without medically confirmed infertility and able to communicate in English or Spanish.

*Data and biospecimen collection*. Research assistants traveled to participants’ homes for the collection of data and biospecimens. Specifically, males completed baseline interviews, followed by a standardized anthropometric assessment for the determination of body mass index (BMI). After completion of the interview, blood collection equipment determined to be free of the environmental chemicals under study was used to obtain 10 cc of blood. Blood samples were transported on ice to the laboratory for processing; 2 mL of serum was used for the analysis of PFCs. Full human subjects approval was obtained from all participating institutions, and all participants provided written informed consent prior to enrollment into the study.

After enrollment in the study, men provided two semen samples approximately 1 month apart. Specimens were obtained via masturbation without the use of lubricants. Following a 2-day abstinence period, men obtained specimens via masturbation, without the use of lubricants, using at-home collection kits (Royster et al. 2000). Each kit included a glass collection jar with an attached button thermometer to monitor temperature every half hour throughout the process, a glass sperm migration straw (Vitrotubes 3520; VitroCom Inc., Mountain Lakes, NJ) containing hyaluronic acid and plugged at one end, and packing materials for shipping. Men were instructed to collect the semen sample in the jar, place the sperm migration straw into the jar (as an exploratory marker of sperm motility and viability at the time of collection), and to record on the label the time of last ejaculation and any spillage. When the specimen was ready for shipment, the men called a toll-free hotline to report sending their semen samples. Specimens were shipped in insulated shipping containers containing ice packs via overnight carrier to the andrology laboratory at the National Institute for Occupational Safety and Health (Cincinnati, OH).

*Semen analysis*. Upon receipt, all semen samples were found to be within acceptable temperature limits and were thus used for analysis. Samples were warmed to 37°C and volume was measured to the nearest 0.1 cc. Established laboratory protocols that include ongoing quality assurance and control procedures (American Society of Andrology 1996) were used to assess 35 semen parameters for the baseline semen samples, including 5 general characteristics (volume, straw distance, sperm concentration, total count, hypo-osmotic swollen), 8 motility measures, 6 sperm head measures, 12 individual and 2 summary morphology measures, and 2 sperm chromatin stability measures.

Sperm motility was assessed using the HTM-IVOS computer assisted semen analysis system (CASA), and sperm concentration was assessed using the IVOS system and the IDENT™ stain (all from Hamilton Thorne Biosciences, Beverly, MA). Microscope slides were prepared for sperm morphometry. Slides for morphology assessments were prepared by Fertility Solutions® (Cleveland, OH). Sperm viability was determined by hypoosmotic swelling (HOS assay) (Jevendran et al. 1992; Schrader et al. 1990). The migration straw was used so the lab could microscopically assess the distance the vanguard sperm traveled to the nearest millimeter, which indicated sperm motility at the time of collection, in light of using next-day analysis (Turner and Schrader 2006). Although some sperm survive past 24 hr (Stovall et al. 1994) and refrigerated samples maintain sperm chromatin structure (Morris et al. 2003), our next-day motility and straw end points were exploratory, given the absence of established validity for interpreting findings as with clinical semen analysis. Sperm morphometry was conducted using the IVOS METRIX system (Hamilton Thorne Biosciences), and morphology was assessed using both traditional [World Health Organization (WHO) 1992] and strict (Rothmann et al. 2013) classifications. An aliquot of whole semen was diluted in TNE buffer and frozen for the sperm chromatin stability assay (SCSA) (Evenson et al. 2002). SCSA® analysis was conducted by SCSA Diagnostics (Brookings, SD) using a Coulter Epics Elite Flow Cytometer (Coulter, Miami, FL). The SCSA® assay measures sperm DNA damage, which is then quantified as the percentage of separated or damaged DNA (DNA fragmentation index; DFI) and the percentage of highly immature sperm nuclei with abnormal proteins (high stainability) (Evenson 2013). A DFI of 25% is associated with diminished fecundity and fertility (Spanò et al. 2000), as is a high stainability of ≈ 35% (Ménézo et al. 2007).

The second semen sample was obtained to corroborate azoospermia observed in the first sample, after which the male was advised to seek clinical care. An abbreviated semen analysis was performed on the second sample (i.e., volume, concentration, next-day motility, and sperm head morphology).

*Toxicologic analysis*. All analyses were conducted by the Division of Laboratory Sciences at the National Center for Environmental Health, Centers for Disease Control and Prevention (Atlanta, GA), using established protocols for the quantification of seven PFCs: 2-(*N*-ethyl-perfluorooctane sulfonamido) acetate (Et-PFOSA-AcOH), 2-(*N*-methyl-perfluorooctane sulfonamido) acetate (Me-PFOSA-AcOH), perfluorodecanoate (PFDeA), perfluorononanoate (PFNA), perfluorooctane sulfonamide (PFOSA), perfluorooctane sulfonate (PFOS), and perfluorooctanoic acid (PFOA). Quantification was performed using isotope dilution high-performance liquid-chromatography–tandem mass spectrometry and established operating procedures (Kato et al. 2011; Kuklenyik et al. 2005). All concentrations are reported in nanograms per milliliter. We used machine-observed concentrations without substituting concentrations below limits of detection (LODs), consistent with contemporary methods aimed at minimizing associated bias (Richardson and Ciampi 2003; Schisterman et al. 2006). Serum cotinine was quantified (nanograms per milliliter) using liquid chromatography-isotope dilution tandem mass spectrometry (Bernert et al. 1997).

*Statistical analysis*. In the descriptive phase of analysis, we assessed geometric means (GMs) and 95% confidence intervals (CIs) for PFCs by site using the nonparametric Wilcoxon test. In the analytic phase, we used linear mixed models to estimate the difference in each semen quality parameter associated with a one-unit change in the natural log (ln)-transformed concentration after adding 1 to each PFC concentration. This method accounts for the correlation stemming from the use of up to two semen samples per male participant for the select end points measured in both samples (i.e., volume, concentration, next-day motility, and sperm head morphology). Of the 473 men, 378 (80%) provided two semen samples. We ran separate models for each PFC and semen parameter, and estimated beta coefficients (β) and 95% CIs for each model. Specifically, beta coefficients denoted the difference in each semen outcome per unit increase in each PFC. We adjusted *a priori* for age (years), BMI (weight in kilograms divided by height in meters squared), smoking (serum cotinine > 40.35 ng/mL or active smoking), abstinence time (days), sample age (hours), and study site (Carlsen et al. 2004; Jeemon et al. 2010; Jensen et al. 1998; Li et al. 2011; Ramlau-Hansen et al. 2007; Sadeu et al. 2010; Schmid et al. 2013). We conducted sensitivity analyses using Box-Cox analysis to determine the optimal transformation for each semen variable. We found that semen end points required ln transformation (*n* = 14), cubic root transformation (*n* = 6), or no (*n* = 14) transformation using the Shapiro-Wilk *W* statistic to assess all semen quality end points (Handelsman 2002). We also visually assessed the residual plots to affirm normality assumptions. Consistent with the exploratory nature of this work in light of limited data, we did not adjust for multiple comparisons. *p*-Values *<* 0.05 were considered statistically significant.

## Results

A serum sample and at least one semen sample were available for 462 (92%) men. Eleven men had no serum sample, 26 had no semen sample, and 2 men had neither sample. The study cohort comprised mostly white non-Hispanic college-educated men with a mean (± SD) age of 31.8 ± 4.9 years and a mean BMI of 29.8 ± 5.6, with no significant differences by enrollment site (Table 1). Many of the men (57%) had previously fathered a pregnancy and few (17%) were current smokers. Mean (± SD) abstinence times for semen samples one and two were 4.0 ± 4.5 and 4.3 ± 5.6 days, respectively. Only 2 men (0.4%) reported an abstinence time *<* 2 days for the initial sample, as did 10 men (2.7%) who provided a second sample.

**Table 1:** Description of male partners in the cohort by study site: the LIFE Study.

Characteristic	Michigan(*n *= 96)	Texas(*n *= 366)	Total(*n *= 462)
Nonwhite race/ethnicity	9 (9)	42 (12)	51 (11)
≤ High school education	8 (8)	28 (8)	36 (8)
No health insurance	10 (10)	28 (8)	38 (8)
Never fathered a pregnancy	47 (49)	193 (53)	240 (52)
Current smoker (cotinine > 40.35 ng/mL)	18 (19)	66 (18)	84 (18)
Age (years)	32.1 ± 4.5	31.7 ± 4.9	31.8 ± 4.9
BMI (kg/m^2^)	29.7 ± 5.4	29.9 ± 5.8	29.8 ± 5.7
Abstinence time (days)	4.4 ± 4.0	4.0 ± 5.2	4.1 ± 5.0
Sample age (hours)	28.5 ± 10.0	27.8 ± 8.2	28.0 ± 8.6
Data are *n* (%) or mean ± SD. None of the values were statistically significant; all *p*-values (from chi-square test for categorical characteristics and Wilcoxon rank sum test for continuous characteristics) comparing the two sites were > 0.05.			

Table 2 presents the distributions for the seven PFCs by research site. Most of the chemicals were readily detected in men’s serum except for Et-PFOSA-AcOH and PFOSA, for which 97% and 84% of concentrations, respectively, were *<* LOD. We observed no statistically significant differences in PFC concentrations between men who did or did not provide a semen sample, except for a higher PFNA concentration in men without a semen sample (GM = 1.82 ng/mL; 95% CI: 1.52, 2.18) compared with those who provided a semen sample (GM = 1.50 ng/mL; 95% CI: 1.43, 1.58) (see Supplemental Material, Table S1). Correlation coefficients between PFCs were low (range, 0.02–0.6), except for PFNA and PFDeA (*r* = 0.8), PFNA and PFOS (*r* = 0.7), and PFOS and PFDeA (*r* = 0.7).

**Table 2:** Distribution of serum PFC concentrations in male partners by availability of semen samples: the LIFE Study.

PFC (ng/mL)	Percent < LOD	Michigan (*n *= 96)		Texas (*n *= 366)	
		GM (95% CI)	Median (IQR)	GM (95% CI)	Median (IQR)
Et-PFOSA-AcOH	97	0.12 (0.10, 0.14)	0 (0, 0.1)	0.12 (0.10, 0.13)	0 (0, 0)
Me-PFOSA-AcOH	22	0.47 (0.40, 0.54)	0.4 (0.3, 0.7)	0.29 (0.26, 0.31)	0.25 (0.1, 0.5)
PFDeA	5	0.31 (0.28, 0.35)	0.3 (0.2, 0.4)	0.47 (0.45, 0.50)	0.5 (0.3, 0.6)
PFNA	1	0.96 (0.84, 1.11)	1.0 (0.75, 1.35)	1.68 (1.61, 1.76)	1.65 (1.2, 2.2)
PFOA	< 1	4.29 (3.86, 4.77)	4.6 (3.0, 6.05)	5.09 (4.86, 5.33)	5.3 (4.1, 6.6)
PFOS	< 1	17.39 (14.94, 20.24)	19.15 (14.65, 25.7)	21.23 (20.07, 22.46)	21.6 (15.8, 29.9)
PFOSA	84	0.13 (0.11, 0.15)	0 (0, 0.1)	0.11 (0.10, 0.12)	0 (0, 0)
IQR, interquartile range. All differences were statistically significant (*p* ≤ 0.02), comparing the two sites. Thirty-nine men were excluded because of missing PFC measurements (*n *= 13) or semen samples (*n *= 2).					

When each PFC and semen parameter were modeled individually, we observed several significant associations, some of which were suggestive of diminished semen quality (Table 3). Et-PFOSA-AcOH was the only PFC not associated with any semen quality parameter; however, data for Et-PFOSA-AcOH are difficult to interpret because concentrations were *<* LOD in 97% of samples. In the primary analysis or without Box-Cox transformation, three semen quality end points were associated with two or more PFCs: *a*) a reduction in the percentage of sperm with coiled tail (PFDeA, PFNA, PFOA, and PFOS); *b*) a reduction in the percentage of sperm with high DNA stainability (MePFOSA-AcOH and PFOSA); and *c*) an increase in the number of immature sperm (MePFOSA-AcOH and PFOSA). Other semen quality parameters were significantly associated with individual PFCs, but without a clear pattern.

**Table 3:** Estimated differences in semen quality parameters associated with a 1-unit increase in ln-transformed serum PFC concentrations: the LIFE Study.

Semen parameter	Et-PFOSA-AcOH β (95% CI)	Me-PFOSA-AcOH β (95% CI )	PFDeAβ (95% CI)	PFNAβ (95% CI)	PFOAβ (95% CI)	PFOSβ (95% CI)	PFOSAβ (95% CI)
General characteristics^*a*^
Semen volume (mL)	–0.452 (–1.837, 0.933)	–0.550 (–1.118, 0.018)	0.630 (–0.195, 1.455)	0.093 (–0.388, 0.574)	–0.092 (–0.457, 0.273)	0.058 (–0.197, 0.312)	–1.433 (–4.626, 1.760)
Sperm viability (%)^*a*^	3.154 (–5.212, 11.520)	–0.342 (–3.932, 3.248)	1.612 (–3.451, 6.675)	1.564 (–1.378, 4.506)	–0.777 (–3.016, 1.463)	–0.056 (–1.621, 1.509)	–5.922 (–25.61, 13.767)
Total sperm count (concentration × 10^6^/mL)	18.873 (–143.522, 181.267)	–9.951 (–77.147, 57.246)	81.003 (–16.059, 178.065)	45.125 (–11.347, 101.598)	0.679 (–42.300, 43.658)	9.741 (–20.243, 39.724)	130.776 (–246.228, 507.780)
Sperm concentration (× 10^6^/mL)	14.848 (–34.366, 64.062)	8.200 (–12.056, 28.456)	–1.063 (–30.457, 28.332)	5.218 (–11.870, 22.306)	0.388 (–12.581, 13.357)	0.025 (–9.018, 9.068)	47.187 (–66.294, 160.668)
Sperm motility^*a*^
Average path velocity (μm/sec)	2.153 (–8.455, 12.761)	–0.106 (–4.641, 4.430)	3.761 (–2.637, 10.158)	2.293 (–1.426, 6.013)	2.223 (–0.603, 5.050)	0.378 (–1.598, 2.355)	9.433 (–15.420, 34.287)
Straight line velocity (μm/sec)	2.380 (–6.296, 11.056)	–0.145 (–3.861, 3.570)	2.947 (–2.293, 8.186)	2.118 (–0.927, 5.162)	1.614 (–0.702, 3.930)	0.646 (–0.972, 2.264)	1.257 (–19.117, 21.632)
Curvilinear velocity (μm/sec)	0.151 (–18.037, 18.340)	–0.664 (–8.453, 7.125)	4.773 (–6.216, 15.762)	3.139 (–3.25, 9.528)	4.982* (0.137, 9.827)	0.293 (–3.101, 3.688)	16.779 (–25.918, 59.475)
Amplitude head displacement (μm)	–0.372 (–1.523, 0.780)	–0.328 (–0.825, 0.168)	0.419 (–0.281, 1.119)	0.176 (–0.232, 0.583)	0.165 (–0.145, 0.475)	–0.086 (–0.303, 0.131)	–0.354 (–3.089, 2.380)
Beat cross frequency (Hz)	2.662 (–3.234, 8.559)	–0.735 (–3.289, 1.819)	0.608 (–2.987, 4.203)	0.542 (–1.549, 2.633)	1.374 (–0.213, 2.961)	–0.136 (–1.249, 0.976)	–3.545 (–17.583, 10.492)
Straightness (%)	7.705 (–8.556, 23.966)	0.117 (–6.898, 7.132)	6.975 (–2.888, 16.839)	4.019 (–1.718, 9.756)	3.736 (–0.627, 8.099)	1.254 (–1.800, 4.308)	13.216 (–25.281, 51.714)
Linearity (%)	7.164 (–3.424, 17.753)	0.420 (–4.168, 5.009)	5.507 (–0.932, 11.946)	3.021 (–0.725, 6.768)	1.570 (–1.286, 4.427)	1.116 (–0.881, 3.112)	11.462 (–13.728, 36.653)
Percent motility (%)	5.915 (–4.500, 16.331)	0.676 (–3.732, 5.085)	3.672 (–2.564, 9.908)	2.516 (–1.108, 6.139)	1.437 (–1.319, 4.192)	1.556 (–0.361, 3.473)	–4.190 (–28.297, 19.918)
Sperm head^*a*^
Length (μm)	–0.145 (–0.396, 0.105)	–0.029 (–0.134, 0.077)	–0.155* (–0.304, –0.006)	–0.064 (–0.151, 0.023)	–0.037 (–0.103, 0.029)	–0.029 (–0.075, 0.017)	–0.509 (–1.085, 0.066)
Area (μm^2^)	–0.335 (–1.096, 0.427)	–0.150 (–0.473, 0.174)	–0.271 (–0.728, 0.186)	–0.201 (–0.467, 0.064)	–0.156 (–0.358, 0.046)	–0.132 (–0.272, 0.009)	–2.295* (–4.052, –0.538)
Width (μm)	0.006 (–0.151, 0.163)	–0.023 (–0.090, 0.043)	0.017 (–0.077, 0.111)	–0.023 (–0.078, 0.031)	–0.026 (–0.068, 0.015)	–0.024 (–0.053, 0.005)	–0.210 (–0.571, 0.152)
Perimeter (μm)	–0.249 (–0.695, 0.197)	–0.068 (–0.256, 0.121)	–0.227 (–0.493, 0.039)	–0.118 (–0.273, 0.037)	–0.066 (–0.184, 0.052)	–0.066 (–0.148, 0.016)	–1.252* (–2.276, –0.228)
Elongation factor (%)	1.476 (–3.295, 6.247)	–0.054 (–2.051, 1.942)	2.122 (–0.706, 4.949)	0.294 (–1.355, 1.942)	–0.203 (–1.454, 1.047)	–0.191 (–1.061, 0.679)	3.121 (–7.753, 13.995)
Acrosome area of head (%)	2.457 (–1.821, 6.735)	1.140 (–0.665, 2.945)	1.367 (–1.190, 3.924)	1.405 (–0.079, 2.889)	1.304* (0.180, 2.428)	0.288 (–0.500, 1.075)	9.785 (–0.047, 19.617)
Straw
Distance (mm)^*b*^	–1.439 (–7.612, 4.734)	0.648 (–2.226, 3.522)	2.727 (–1.166, 6.621)	1.742 (–0.536, 4.019)	–0.001 (–1.674, 1.672)	1.231* (0.089, 2.372)	13.890 (–2.427, 30.206)
Sperm morphology^*b*^
Percent normal, strict criteria^*c*^	4.816 (–4.735, 14.367)	–0.385 (–4.669, 3.900)	4.914 (–0.829, 10.658)	3.897* (0.564, 7.231)	1.916 (–0.561, 4.392)	1.720 (–0.012, 3.452)	18.709 (–4.918, 42.335)
Percent normal, WHO criteria^*d*^	6.096 (–5.728, 17.920)	–0.432 (–5.737, 4.873)	5.799 (–1.313, 12.912)	3.968 (–0.168, 8.104)	1.726 (–1.345, 4.796)	1.835 (–0.312, 3.982)	25.417 (–3.815, 54.648)
Amorphous (%)	–4.101 (–14.484, 6.281)	0.981 (–3.674, 5.635)	–0.435 (–6.698, 5.827)	–1.437 (–5.080, 2.206)	–0.372 (–3.070, 2.327)	–0.259 (–2.150, 1.631)	–10.054 (–35.783, 15.674)
Round (%)	–1.309 (–2.832, 0.213)	–0.056 (–0.740, 0.629)	0.365 (–0.555, 1.285)	0.311 (–0.224, 0.846)	–0.020 (–0.416, 0.377)	0.162 (–0.116, 0.440)	–1.074 (–4.858, 2.711)
Pyriform (%)	–0.248 (–6.234, 5.739)	–0.126 (–2.809, 2.556)	–1.605 (–5.21, 2.000)	–0.012 (–2.113, 2.089)	–0.459 (–2.013, 1.096)	–0.632 (–1.719, 0.456)	–1.376 (–16.212, 13.459)
Bicephalic (%)	–0.186 (–1.800, 1.427)	0.283 (–0.439, 1.006)	0.658 (–0.312, 1.629)	0.169 (–0.397, 0.735)	0.325 (–0.093, 0.743)	0.205 (–0.088, 0.498)	4.127* (0.149, 8.105)
Tapered (%)	–1.337 (–3.844, 1.170)	–0.623 (–1.746, 0.501)	–0.717 (–2.228, 0.795)	–0.051 (–0.932, 0.830)	0.117 (–0.535, 0.769)	0.037 (–0.42, 0.494)	–6.179 (–12.371, 0.014)
Megalo head (%)	–0.732 (–2.528, 1.064)	0.088 (–0.717, 0.893)	–0.632 (–1.713, 0.450)	–0.355 (–0.985, 0.275)	–0.392 (–0.857, 0.073)	–0.257 (–0.584, 0.069)	–1.085 (–5.537, 3.367)
Micro head (%)	–0.743 (–1.865, 0.378)	–0.171 (–0.675, 0.332)	0.114 (–0.563, 0.792)	–0.019 (–0.413, 0.375)	–0.214 (–0.505, 0.077)	–0.084 (–0.288, 0.120)	0.555 (–2.23, 3.340)
Neck or midpiece abnormalities (%)	–5.766 (–15.38, 3.848)	5.011* (0.724, 9.298)	–2.624 (–8.423, 3.175)	–2.462 (–5.833, 0.909)	–1.845 (–4.340, 0.650)	–0.512 (–2.264, 1.240)	–5.048 (–28.909, 18.812)
Coiled tail (%)	–6.898 (–17.585, 3.789)	0.099 (–4.699, 4.898)	–7.603* (–14.014, –1.193)	–4.030* (–7.766, –0.293)	–2.768* (–5.536, 0.000)	–2.274* (–4.209, –0.338)	–9.128 (–35.650, 17.395)
Other tail abnormalities (%)	–0.451 (–4.453, 3.551)	1.440 (–0.348, 3.227)	–0.354 (–2.766, 2.057)	–0.519 (–1.922, 0.885)	–0.112 (–1.151, 0.928)	–0.327 (–1.054, 0.401)	–3.089 (–13.002, 6.824)
Cytoplasmic droplet (%)	–0.949 (–6.000, 4.103)	–1.825 (–4.082, 0.431)	–0.172 (–3.217, 2.873)	0.123 (–1.65, 1.896)	–0.348 (–1.66, 0.963)	–0.407 (–1.326, 0.511)	–9.053 (–21.541, 3.436)
Immature sperm (*n*)	–4.963 (–21.346, 11.419)	18.719** (11.611, 25.827)	3.629 (–6.243, 13.501)	2.610 (–3.135, 8.355)	–0.411 (–4.668, 3.846)	2.095 (–0.880, 5.070)	90.881** (51.266, 130.496)
Sperm chromatin stability^*b*^
DNA fragmentation index (%)	–2.634 (–11.979, 6.710)	1.234 (–2.859, 5.328)	–4.040 (–9.793, 1.712)	–2.878 (–6.155, 0.400)	–0.713 (–3.184, 1.757)	–0.914 (–2.678, 0.849)	–0.899 (–23.035, 21.236)
High DNA stainability (%)	–0.539 (–5.395, 4.317)	–2.552* (–4.665, –0.438)	–0.929 (–3.923, 2.065)	–0.687 (–2.395, 1.020)	–0.115 (–1.399, 1.169)	–0.423 (–1.340, 0.493)	–15.153** (–26.559, –3.747)
Analysis includes 5 azoospermic men but excludes 39 men who had missing values for PFCs (*n *= 11), missing semen samples (*n *= 26), or both (*n *= 2). Fixed-effects and mixed-effects models were used to analyze semen parameters with one and two measurements, respectively. PFC concentrations were ln-transformed and adjusted for age (years), BMI (kg/m^2^), serum cotinine (active smoking yes/no), abstinence (days), sample age (hours), and research site (Texas/Michigan).^***a***^Assessed in both semen samples. ^***b***^Assessed by SCSA^®^ analysis only in the baseline semen sample. ^***c***^Rothmann et al. (2013). ^***d***^WHO (1992). *p < 0.05. **p < 0.01.							

Of the seven PFCs examined, PFOSA, MePFOSA-AcOH, and PFOA were most often observed to be associated with semen quality end points, with 5, 3, and 2 separate associations, respectively. Specifically, a 1-unit increase in ln-transformed PFOSA was associated with smaller sperm head area (β = –2.295; 95% CI: –4.052, –0.538), smaller sperm perimeter (β = –1.252; 95% CI: –2.276, –0.228), lower percentage of sperm with high DNA stainability (β = –15.153; 95% CI: –26.559, –3.747), higher percentage of bicephalic sperm (β = 4.127; 95% CI: 0.149, 8.105), and higher numbers of immature sperm (β = 90.881; 95% CI: 51.266, 130.496). Me-PFOSA-AcOH was associated with a higher percentage of sperm with neck/midpiece abnormalities (β = 5.011; 95% CI: 0.724, 9.298), higher numbers of immature sperm (β = 18.719; 95% CI: 11.611, 25.827), and a lower percentage with high DNA stainability (β = –2.552; 95% CI: –4.665, –0.438). In sensitivity analyses, 16 of 17 significant (*p <* 0.05) associations were observed, 15 of which were observed in the primary analysis (i.e., volume was not significant in the sensitivity analysis). Complete sensitivity results are provided in Supplemental Material, Table S2.

## Discussion

Findings of the present study suggest that select PFCs at environmentally relevant concentrations may be adversely associated with semen quality, with the exception of Et-PFOSA-AcOH, for which only 3% of concentrations were > LOD. Associations suggestive of diminished semen quality included differences in sperm head (increased bicephalic) and morphology (increased immature sperm). Interpretation of these findings is uncertain, given the lack of well-established clinical norms for many individual parameters and because of reliance on next-day semen analysis and possible spurious associations. Morphologic sperm characteristics, including those involving the tail, may provide information about underlying mechanisms during the maturation process and eventual fertility, particularly in the context of other biochemical markers (Durutovic et al. 2013). In addition, each of these PFCs was associated with other semen characteristics, underscoring their varying patterns. Of the PFCs tested, PFOSA, a precursor to PFOS, was significantly associated with the most semen parameters, including an increased percentage of bicephalic sperm and the number of immature sperm, both suggestive of diminished semen quality. However, we recognize the absence of established levels between normal sperm head morphology and genetic quality of spermatozoa (Ryu et al. 2001; Simon and Lewis 2011), limiting further speculation regarding our findings. We did not observe evidence of sperm DNA fragmentation on the basis of SCSA findings, which indicated a lower percentage of damaged sperm irrespective of PFC. It is important to note that only 16% of PFOSA concentrations were above the LOD, although quantification of PFCs was blinded to semen quality. The low prevalence of such exposure, with most of the measured concentrations below the LOD, is consistent with PFOSA’s discontinued production in the United States. The lack of consistent associations with motility—other than two positive associations for percent motility and straw distance—may reflect our reliance on next-day analysis, which provides only an exploratory assessment of viability and motility at the timing of collection absent validation methods.

Our findings suggesting a negative association between select PFCs and sperm morphology are somewhat consistent with those reported for male partners of pregnant women seeking prenatal care (Toft et al. 2012); in that study, a 35% reduction in the proportion of morphologically normal sperm was observed for men in the highest relative to the lowest tertile of PFOS. Because tests for assessing sperm DNA are not equivalent, using the SCSA method in the present study, we were unable to assess earlier findings such as an increased percentage of TUNEL-positive sperm cells for serum PFOA concentrations, as reported in two of three subgroups of male participants in the INUENDO cohort. We also corroborated the absence of an association between PFCs and the percentage of DNA fragmentation when using SCSA techniques (Specht et al. 2012). Of note was our observation of a negative association between Me-PFOSA-AcOH and PFOSA and the percentage of high DNA stainability, suggesting fewer sperm with immature chromatin. The absence of previous research focusing on this outcome precludes a more complete interpretation of this finding.

Median concentrations of PFCs for men participating in the LIFE Study were comparable to those reported in five earlier studies (see Supplemental Material, Table S3), despite differences in sampling frameworks ranging from medical clinics (Raymer et al. 2012; Specht et al. 2012; Toft et al. 2012) to the general population (Joensen et al. 2009, 2013). In addition to recruiting men from targeted geographic areas who were not seeking medical care, our findings are further strengthened by the availability of both PFC and semen quality data for 92% of the cohort, the analysis of all semen samples provided by men, and our direct measurement of BMI and serum cotinine. PFC concentrations of participants in the LIFE Study are comparable to those reported in the National Health and Nutrition Examination Survey (NHANES) despite the older age range of NHANES participants (Centers for Disease Control and Prevention 2009). However, GM concentrations of PFOS were higher in the LIFE Study than in NHANES (20.52; 95% CI: 19.47, 21.63 and 16.3; 95% CI: 15.0, 17.70, respectively).

Important limitations need to be considered in the interpretation of our results, most notably the use of a next-day semen analysis. There has been limited study of home versus clinical semen collection emphasizing the exploratory nature of next-day analysis. Our motility findings have uncertain meaning and cannot be directly compared with clinical semen analysis. Previous researchers have successfully used home semen collection for the assessment of environmental exposures, but they did not report motility findings (Luben et al. 2007; Olshan et al. 2007). These authors did, however, note that all returned samples retained motile and viable sperm. In the preent study, we used the straw measure as a similar global measure. A second limitation of our study is the timing of exposure relative to semen collection, which makes it difficult to identify the sensitive window; this is an issue relevant for all past research as well. Although the long half-lives of PFCs most likely precede the relevant sensitive window (≈ 72 days) for spermatogenesis and the hormonal milieu for the our analysis of semen samples, we cannot rule out possible *in utero* exposures that result in epigenetic spermatozoa defects (Aitken 2010). However, we are unable to address the exact timing of exposure relevant for semen quality, such as the *in utero* window as recently reported by Vested et al. (2013). A third limitation of the present study is the absence of any reproductive hormone measurements, particularly in light of earlier reports of both a negative relation between serum PFOS and testosterone (Joensen et al. 2013) and a positive relation for plasma PFOA and PFOS (Raymer et al. 2012). Finally, we cannot rule out chance findings given our exploratory analysis that comprised 245 comparisons, of which only 7% were significant at *p <* 0.05.

Our findings may have potential implications for male reproductive health or couple fecundity. Semen analysis provides useful information on sperm production, motility, viability, genital tract patency, accessory gland function, and ejaculation capability, but its predictive value for fertility remains limited (Niederberger 2011; Practice Committee of the American Society of Reproductive Medicine 2013) underscoring the need for continued investigation of fecundity biomarkers suitable for population health research.

The mechanisms through which PFC exposure may affect semen quality remain unknown, although several explanations have been suggested including the PFCs’ estrogenic-like properties (Liu et al. 2007) and their ability to alter the hormonal milieu (Damgaard et al. 2002), lipid metabolism (Kennedy et al. 2004), inflammatory processes and responses (Corsini et al. 2012), and reactive oxygen species (Mathur et al. 2008). Irrespective of mechanism(s), findings of the present study support continual efforts aimed at elucidating environmental impacts on semen quality, particularly given increasing global concern about declining male fecundity (Pflieger-Bruss et al. 2004; Priskorn et al. 2012). Despite these concerns, limited research has been conducted to determine the impact of environmental chemicals on human semen quality, resulting in continued reliance on animal findings (Phillips and Tanphaichitr 2008). The highly timed and interrelated nature of spermatogenesis, whereby immature spermatogonia develop into mature spermatids for their eventual release and continued maturation through the epididymis, underscores their vulnerability throughout this sensitive window in relation to environmental influences.

## Conclusions

We found that select PFCs at environmentally relevant concentrations were associated with differences in sperm head, morphology, and DNA characteristics, including differences indicative of higher and lower semen quality. These exploratory findings suggest some deleterious differences in sperm morphology (e.g., immature, bicephalic) but await corroboration. Follow-up investigation of the impact of semen changes on male reproductive health or couple fecundity is needed, including in-depth semen analyses.

## Supplemental Material

(248 KB) PDF
