# Supplementary material for: Perfluorochemicals and Human Semen Quality: The LIFE Study
Source: Environ Health Perspect. 2014 Aug 15;123(1):57–63. doi: 10.1289/ehp.1307621 (PMC4286271; doi:10.1289/ehp.1307621)
Supplement: (248 KB) PDF [file ehp.1307621.s001.508.pdf]

Supplemental Material

Perfluorochemicals and Human Semen Quality: The LIFE Study

Germaine M. Buck Louis, Zhen Chen, Enrique F. Schisterman, Sungduk Kim, Anne M. Sweeney,

Rajeshwari Sundaram, Courtney D. Lynch, Robert E. Gore-Langton, and Dana Boyd Barr

Table of Contents **Page**

Table S1. Geometric mean comparison and ranges of perfluorochemical concentrations by availability of a semen sample, LIFE Study (n=488). 2

Table S2. Estimated change in semen quality parameters after Box-Cox transformation by serum perfluorochemicals, LIFE Study (n=462). 3

Table S3. Comparison of PFC concentrations in LIFE Study male participants with the published literature. 5

Table S1. Geometric mean comparison and ranges of perfluorochemical concentrations by availability of a semen sample, LIFE Study (n=488).

Perfluorochemicals (ng/mL)	With semen sample (n=462) Mean (95% CI)	With semen sample (n=462) Range	No semen sample (n=26) Mean (95% CI)	No semen sample (n=26) Range
Et-PFOSA-AcOH	0.12 (0.11,0.13)	0 - 2.9	0.13 (0.07, 0.22)	0 - 0.5
Me-PFOSA-AcOH	0.32 (0.30,0.34)	0 - 4.6	0.33 (0.24, 0.46)	0 - 1.6
PFDeA	0.44 (0.41,0.46)	0 - 2.5	0.51 (0.39, 0.68)	0 - 4.4
PFNA*	1.50 (1.43,1.58)	0 - 6.7	1.82 (1.52, 2.18)	0.9 - 3.8
PFOA	4.91 (4.71, 5.13)	0 - 15.4	5.33 (4.68, 6.01)	3.1 - 8.2
PFOS	20.37 (19.28, 21.51)	0 - 159	23.52 (19.84, 27.89)	10.0 - 53.4
PFOSA	0.12 (0.11, 0.13)	0 - 0.4	---	0 - 0.1

(---) denotes that the concentrations were not appropriate for estimation of geometric means as 26 men had PFOSA concentrations either at 0 or at 0.1.

*$p<0.05$ comparing mean PFC concentrations between men with and without semen samples.

Table S2. Estimated change in semen quality parameters after Box-Cox transformation by serum perfluorochemicals, LIFE Study (n=462).

Characteristic	Et-PFOSA-AcOH β (95%CI)	Me-PFOSA-AcOH β (95%CI)	PFDeA β (95%CI)	PFNA β (95%CI)	PFOA β (95%CI)	PFOS β (95%CI)	PFOSA β (95%CI)
General characteristics[a]							
Volume (mL)	-0.168 (-0.79, 0.454)	**-0.267*** **(-0.522, -0.012)**	0.262 (-0.108, 0.633)	0.012 (-0.204, 0.228)	-0.049 (-0.213, 0.114)	-0.002 (-0.116, 0.112)	-0.852 (-2.285, 0.581)
Viability (%)[a]	3.154 (-5.212, 11.52)	-0.342 (-3.932, 3.248)	1.612 (-3.451, 6.675)	1.564 (-1.378, 4.506)	-0.777 (-3.016, 1.463)	-0.056 (-1.621, 1.509)	-5.922 (-25.61, 13.767)
Total count (concentration x10^6/mL)	1.018 (-3.832, 5.867)	-1.106 (-3.103, 0.89)	1.818 (-1.077, 4.712)	0.972 (-0.711, 2.656)	-0.137 (-1.417, 1.142)	-0.048 (-0.94, 0.845)	1.435 (-9.775, 12.644)
Sperm concentration (x10^6/mL)	1.210 (-1.932, 4.351)	0.009 (-1.28, 1.298)	0.130 (-1.741, 2.001)	0.441 (-0.646, 1.528)	-0.010 (-0.835, 0.816)	-0.093 (-0.668, 0.482)	2.897 (-4.311, 10.105)
Sperm motility[a]							
Percent motility (%)	1.194 (-1.157, 3.546)	0.063 (-0.933, 1.059)	1.405 (0, 2.809)	0.767 (-0.05, 1.584)	0.232 (-0.391, 0.855)	0.214 (-0.22, 0.648)	0.711 (-4.738, 6.16)
Sperm head[a]							
Length (μm)	-0.025 (-0.068, 0.017)	-0.005 (-0.023, 0.013)	**-0.026*** **(-0.051, -0.001)**	-0.011 (-0.026, 0.004)	-0.006 (-0.017, 0.005)	-0.005 (-0.013, 0.003)	-0.089 (-0.186, 0.009)
Straw distance (mm)[b]	-0.057 (-0.59, 0.476)	-0.001 (-0.249, 0.247)	0.284 (-0.052, 0.619)	0.139 (-0.058, 0.335)	-0.013 (-0.157, 0.131)	**0.102*** **(0.003, 0.2)**	1.103 (-0.306, 2.512)
Morphology[b]							
Amorphous (%)	-0.275 (-1.23, 0.679)	-0.003 (-0.431, 0.425)	-0.004 (-0.58, 0.571)	-0.130 (-0.464, 0.205)	-0.047 (-0.295, 0.201)	-0.029 (-0.202, 0.145)	-1.459 (-3.821, 0.903)
Round (%)	-0.480 (-1.008, 0.047)	-0.070 (-0.307, 0.167)	0.091 (-0.228, 0.411)	0.109 (-0.076, 0.295)	-0.015 (-0.153, 0.122)	0.034 (-0.062, 0.13)	-1.001 (-2.31, 0.308)
Pyriform (%)	0.153 (-0.679, 0.985)	0.025 (-0.348, 0.397)	-0.423 (-0.923, 0.076)	-0.121 (-0.413, 0.171)	-0.098 (-0.314, 0.118)	-0.068 (-0.219, 0.083)	-0.277 (-2.338, 1.785)
Bicephalic (%)	0.132 (-0.404, 0.667)	0.130 (-0.109, 0.37)	0.219 (-0.103, 0.541)	0.034 (-0.154, 0.222)	0.093 (-0.046, 0.232)	0.061 (-0.037, 0.158)	**1.636*** **(0.318, 2.953)**
Taper (%)	-0.209 (-0.863, 0.445)	-0.102 (-0.395, 0.191)	-0.245 (-0.639, 0.149)	-0.007 (-0.237, 0.222)	0.075 (-0.095, 0.245)	0.037 (-0.082, 0.156)	-1.560 (-3.174, 0.054)
Megalo head (%)	-0.140 (-0.644, 0.363)	-0.023 (-0.249, 0.203)	-0.159 (-0.462, 0.144)	-0.089 (-0.265, 0.088)	-0.127 (-0.257, 0.004)	-0.068 (-0.159, 0.023)	-0.652 (-1.899, 0.594)
Micro head (%)	-0.314 (-0.75, 0.121)	-0.060 (-0.255, 0.136)	0.058 (-0.206, 0.321)	-0.002 (-0.156, 0.151)	-0.079 (-0.192, 0.034)	-0.028 (-0.108, 0.051)	0.370 (-0.711, 1.452)

Characteristic	Et-PFOSA-AcOH β (95%CI)	Me-PFOSA-AcOH β (95%CI)	PFDeA β (95%CI)	PFNA β (95%CI)	PFOA β (95%CI)	PFOS β (95%CI)	PFOSA β (95%CI)
Neck or midpiece abnormalities (%)	-0.258 (-0.744, 0.227)	**0.231* (0.015, 0.447)**	-0.122 (-0.414, 0.171)	-0.116 (-0.286, 0.054)	-0.106 (-0.232, 0.019)	-0.024 (-0.112, 0.065)	-0.304 (-1.507, 0.9)
Coiled tail (%)	-0.367 (-0.939, 0.204)	0.018 (-0.238, 0.275)	**-0.454** (-0.796, -0.112)**	**-0.242* (-0.441, -0.042)**	**-0.171* (-0.319, -0.023)**	**-0.140** (-0.244, -0.037)**	-0.292 (-1.711, 1.127)
Other tail abnormalities (%)	0.057 (-0.509, 0.622)	0.193 (-0.06, 0.445)	-0.032 (-0.373, 0.309)	-0.105 (-0.303, 0.093)	-0.020 (-0.166, 0.127)	-0.061 (-0.164, 0.042)	-0.114 (-1.515, 1.287)
Cytoplasmic droplet (%)	-0.321 (-1.476, 0.834)	-0.489 (-1.004, 0.027)	-0.087 (-0.784, 0.609)	-0.078 (-0.483, 0.327)	-0.128 (-0.428, 0.171)	-0.158 (-0.368, 0.052)	-2.342 (-5.195, 0.512)
Immature sperm (#)	0.020 (-0.82, 0.861)	0.234 (-0.142, 0.61)	0.168 (-0.338, 0.674)	0.136 (-0.158, 0.431)	0.028 (-0.19, 0.246)	0.005 (-0.148, 0.158)	0.774 (-1.308, 2.855)
Sperm chromatin stability assay[b]							
DNA fragmentation index (%)	-0.269 (-0.898, 0.36)	0.045 (-0.23, 0.321)	-0.264 (-0.651, 0.124)	-0.203 (-0.424, 0.018)	-0.060 (-0.226, 0.106)	-0.072 (-0.191, 0.047)	0.031 (-1.461, 1.522)
High DNA stainability (%)	0.047 (-0.531, 0.625)	**-0.271* (-0.522, -0.019)**	-0.116 (-0.472, 0.24)	-0.079 (-0.283, 0.124)	-0.007 (-0.16, 0.146)	-0.048 (-0.157, 0.061)	**-1.856** (-3.212, -0.499**)

NOTE: The analysis excluded 39 men missing either PFCs (n=11), semen samples (n=26) or both (n=2). Fixed and mixed effects models were used for the analysis of semen parameters with one and two measurements, respectively. PFC concentrations were natural logarithm transformed and adjusted for age (continuous), BMI (continuous), active smoking (yes/no), abstinence (#days), sample age (#hours), and research site (Texas/Michigan). Natural logarithm transformation was undertaken for length, straw distance, round, pyriform, bicephalic, taper, megalo head, micro head, neck or midpiece abnormalities, coiled tail, other tail abnormalities, immature sperm, DNA fragmentation index, and high DNA stainability. The cubic-root transformation was undertaken for volume, total count, sperm concentration, percent motility, amorphous, and cytoplasmic droplet. Strict criteria was transformed as (y^0.7 -1)/0.7. The 14 semen endpoints not requiring transformation are excluded from presentation in this stable.

[a]Assessed in both semen samples. [b]Assessed only in the baseline semen sample.

*p<0.05, **p<0.01.

β, beta coefficient; CI, confidence interval.

Table S3. Comparison of PFC concentrations in LIFE Study male participants with the published literature.

Perfluorcompound	Joensen et al. 2009 n=105 Median (ng/ml)	Toft et al. 2012[a] n=588 Median (ng/ml)	Specht et al. 2012[a] Ukraine n=208 Poland n=197 Greenland n=199 Median (ng/ml)	Raymer et al. 2012 n=252 Median (ng/ml)	Joensen et al. 2013 n=247 Mean (ng/ml)	Louis et al. 2013 n=462 Median (ng/ml)
Et-PFOSA-AcOH	--	--	--	--	--	0.0
Me-PFOSA-AcOH	--	--	--	--	--	0.3
PFDA	0.9	--	0.2<br>0.4<br>0.9	--	0.38	--
PFDeA	--	--	--	--	--	0.4
PFDoA	0.08	--	--	--	--	--
PFDoDA	--	--	0.1<br>0.1<br>0.1	--	--	--
PFHpA	0.2	--	--	--	--	--
PFHpS	--	--	--	--	0.29	
PFHxS	6.6	1.1	0.3<br>1.2<br>2.2	--	0.81	--
PFNA	0.8	1.2	1.0<br>1.2<br>1.4	--	1.23	1.5
PFOA	4.9	3.8	1.3<br>4.8<br>4.5	9.2	3.46	5.2
PFOS	24.5	18.4	7.6<br>18.5<br>44.7	32.3	8.46	20.4
PFOSA	0.06	--	--	--	--	0.0
PFTrA	0.0	--	--	--	--	--
PFUnA	0.1	--	0.3<br>0.3<br>1.3	--	--	--

NOTE: All PFCs were measured in serum except for Raymer et al. 2012, where plasma was used.

[a]Utilized the INUENDO Cohort comprising men from Ukraine, Poland and Greenland. Concentrations only reported for individual countries by Olmer Specht et al.
